# Nutrients and Pharmaceuticals Structure Bacterial Core Communities in Urban and Montane Stream Biofilms

**DOI:** 10.3389/fmicb.2020.526545

**Published:** 2020-10-15

**Authors:** Elizabeth M. Ogata, Michelle A. Baker, Emma J. Rosi, Trevor B. Smart, Donald Long, Zachary T. Aanderud

**Affiliations:** ^1^Department of Biology and Ecology Center, Utah State University, Logan, UT, United States; ^2^Cary Institute of Ecosystem Studies, Millbrook, NY, United States; ^3^Department of Plant and Wildlife Sciences, Brigham Young University, Provo, UT, United States; ^4^Department of Biology, Southern Utah University, Cedar City, UT, United States

**Keywords:** water quality, stream biofilms, nutrients, pharmaceuticals, bacteria, contaminant diffusing substrate

## Abstract

Bacteria in stream biofilms contribute to stream biogeochemical processes and are potentially sensitive to the substantial levels of pollution entering urban streams. To examine the effects of contaminants on stream biofilm bacteria *in situ*, we exposed growing biofilms to experimental additions of nutrients [nitrogen (N), phosphorus (P), and iron (Fe)], pharmaceuticals (caffeine and diphenhydramine), nutrients plus pharmaceuticals, or no contaminants using contaminant exposure substrates (CES) in three catchments in northern Utah. We performed our study at montane and urban sites to examine the influence of existing pollution on biofilm response. We identified bacterial core communities (core) for each contaminant treatment at each land-use type (e.g., nutrient addition montane bacterial core, nutrient addition urban bacterial core, pharmaceutical addition montane bacterial core) by selecting all taxa found in at least 75% of the samples belonging to each specific grouping. Montane and urban land-use distinguished bacterial cores, while nutrients and pharmaceuticals had subtle, but nonetheless distinct effects. Nutrients enhanced the dominance of already abundant copiotrophs [i.e., Pseudomonadaceae (Gammaproteobacteria) and Comamonadaceae (Betaproteobacteria)] within bacterial cores at montane and urban sites. In contrast, pharmaceuticals fostered species-rich bacterial cores containing unique contaminant-degrading taxa within Pseudomonadaceae and Anaerolineaceae (Chloroflexi). Surprisingly, even at urban sites containing ambient pharmaceutical pollution, pharmaceutical additions increased bacterial core richness, specifically within DR-16 (Betaproteobacteria), WCHB1-32 (Bacteroidetes), and Leptotrichiaceae (Fusobacteria). Nutrients exerted greater selective force than pharmaceuticals in nutrient plus pharmaceutical addition treatments, creating bacterial cores more closely resembling those under nutrient rather than pharmaceutical addition, and promoting unique Oscillatoriales (Cyanobacteria) taxa in urban streams. Our results show that additions of N, P, and Fe intensified the dominance of already abundant copiotrophs, while additions of caffeine and diphenhydramine enabled unique taxa associated with contaminant degradation to participate in bacterial cores. Further, biofilm bacteria at urban sites remained sensitive to pharmaceuticals commonly present in waters, suggesting a dynamic interplay among pharmaceutical pollution, bacterial diversity, and contaminant degradation.

## Introduction

Biofilms house the primary form of bacterial life in freshwater streams (Costerton et al., 1978; Geesey et al., 1978; Lock et al., 1984; Battin et al., 2016). Biofilms form from single or co-aggregated microbial cells attaching to stream benthic surfaces, forming chain-like microcolonies that coalesce into larger and more complex communities of bacteria, archaea, algae, fungi, protozoa, and viruses embedded in a polysaccharide extracellular matrix (Battin et al., 2007, 2016; Besemer et al., 2012; Besemer, 2015). Upstream biological propagules of dispersing bacteria enter the water column from soils and tributaries within the surrounding catchment and contribute to burgeoning biofilms. Typically, stream biofilms are dominated by taxa from three main phyla: Proteobacteria, Bacteroidetes, and Cyanobacteria (Besemer, 2015) and natural physiochemical water conditions such as pH, water temperature, and dissolved organic carbon concentrations influence biofilm bacterial composition (Besemer, 2015). For example, stream bacterial communities were strongly correlated with stream water pH across the 3000-ha Hubbard Brook watershed in New Hampshire, United States, where an increase in stream water pH was associated with an increase in the relative abundance of Proteobacteria and decrease in Acidobacteria (Fierer et al., 2007b). In addition to natural river conditions, contaminants associated with human activities may dramatically alter biofilm bacterial biomass and composition (Tank and Dodds, 2003; Rosi-Marshall et al., 2013; Wang et al., 2018).

Nutrients entering streams and rivers through wastewater, fertilizers, and atmospheric deposition (Carey et al., 2013) are major freshwater contaminants with potential to alter bacterial communities. Human activities have dramatically enhanced global nitrogen (N) and phosphorus (P) cycles (Falkowski et al., 2000), with global riverine exports of dissolved inorganic N and P estimated to have increased by about 30% between 1970 and 2000 (Seitzinger et al., 2010). Nutrient pollution is recognized as a leading contributor to water quality impairment (Withers et al., 2014) and can have important consequences for stream bacteria. The growth of aquatic heterotrophs and oxygenic phototrophs is often limited by the availability of N and P, and to a lesser extent iron (Fe) required for photosynthesis (Van Horn et al., 2011; Larson et al., 2018). Further, nutrients may alter bacterial composition as taxa differ in their ability to thrive under various nutrient conditions (Fierer et al., 2007a). Among Proteobacteria, one of the major constituents of stream biofilms, Betaproteobacteria and Gammaproteobacteria contain many opportunistic or copiotroph species that thrive in nutrient-rich conditions (Shi et al., 1999; Fierer et al., 2007a), while Alphaproteobacteria may thrive in oligotrophic waters (Pinhassi and Berman, 2003). In heterotrophic biofilms grown in stream mesocosms, a 10-fold increase in nutrient concentrations led to a 20-fold rise in biomass and enhanced the abundance of copiotrophic heterotrophs such as Betaproteobacteria and Gammaproteobacteria taxa (Van Horn et al., 2011). Urban nutrient run-off in the cities surrounding the Jialing River, China, has dramatically altered biofilms with elevated levels of P, nitrate, and Fe explaining 63.0% of the variation within bacterial communities (Wang et al., 2018).

Like nutrients, pharmaceuticals are ubiquitous in human impacted surface waters (Kolpin et al., 2002; Pal et al., 2010) and can alter biofilm bacterial biomass and composition (Lawrence et al., 2005; Rosi-Marshall et al., 2013; Rosi et al., 2018). Pharmaceutical pollution is also a global issue: pharmaceuticals or their transformation products are at least detectable in many aquatic ecosystems of seventy-one countries on all continents (aus der Beek et al., 2016). Although typically detected at trace concentrations in freshwaters (ng L^–1^ to μg L^–1^; Kolpin et al., 2002; Pal et al., 2010), many pharmaceuticals are designed to be biologically active at even low doses and consequently, are expected to affect non-target organisms, such as bacteria. Caffeine, a stimulant found in a variety of beverages (e.g., coffee, tea, soft drinks, and energy drinks) and medications and diphenhydramine, an antihistamine, are both particularly prevalent pharmaceutical pollutants in freshwaters (Kolpin et al., 2002) with both altering biofilm bacteria. Exposure to high experimental caffeine and diphenhydramine additions decreased respiration of heterotrophic biofilms by 53 and 63%, respectively (Rosi-Marshall et al., 2013). The same study also found that diphenhydramine reduced the relative abundance of Flavobacterium, a common constituent of stream biofilms (Besemer et al., 2012), but increased the abundance of durable *Pseudomonas* species (Gammaproteobacteria), a genus known for its ability to degrade high molecular weight organic compounds (Dastidar et al., 1976; Palleroni, 2010; Rosi-Marshall et al., 2013), suggesting that pharmaceuticals may have negative effects on sensitive taxa but positive effects on taxa capable of degrading recalcitrant compounds. Indeed, caffeine, which may serve as the sole carbon (C) and N source for certain bacteria, increased bacterial biomass and biofilm thickness by over 30% in biofilms from the Saskatchewan River, Canada (Lawrence et al., 2005). Stream bacterial communities may even respond to pharmaceutical pollution by demonstrating enhanced contaminant-degradation potential; the abundance of xenobiotic metabolic genes within bacterial communities increased with the highly-urbanized Jialing River, China (Wang et al., 2018). Given the ubiquitous nature and potential influence of pharmaceuticals on stream biofilms, research to understand the effects of pharmaceuticals such as caffeine and diphenhydramine can enhance our understanding of anthropogenic effects on stream ecosystems.

Contaminant exposure substrates (CES; Rosi-Marshall et al., 2013; Costello et al., 2016) can be used to examine the effects of nutrients, pharmaceuticals, and other contaminants on stream biofilm bacteria *in situ*. CES employ a similar design as nutrient diffusing substrates (NDS) which are widely used in stream ecology to examine nutrient limitation (Tank et al., 2007; Capps et al., 2011). These devices consist of a small container filled with unamended (control) or contaminant-amended agar (contaminant treatment) capped with a porous surface such as a fritted glass disk or cellulose sponge (Tank et al., 2007; Capps et al., 2011). CES are incubated in the stream for 2–3 weeks and biofilms which colonize the porous surfaces are exposed to contaminants diffusing out of the agar. Contaminant effects on biofilm biomass, activity, or community composition can be assessed by comparing biofilms on control and contaminant treatment CES. CES provide ecologically-relevant results as studies are conducted *in situ*. Further, the method is cost-effective and easily replicated, enabling the inclusion of multiple contaminant treatments and study sites. Importantly, contaminant diffusion rates typically decline throughout the in-stream incubation and may be influenced multiple factors such as temperature, stream flow, and water column contaminant concentrations (Shaw et al., 2015). In addition, the composition of biofilms grown on CES may differ from natural biofilms, as CES biofilms develop over only 2–3 weeks on porous surfaces which are structurally homogenous in comparison to natural stream surfaces (Hoellein et al., 2010). Thus, CES may be less appropriate for determining specific ecotoxicology thresholds but can play an important role in detecting contaminant effects on stream biofilm growth, activity, and composition.

To more fully understand the impact of human activities on stream biofilm bacterial community composition, we used CES to assess *in situ* responses of stream biofilm bacteria to nutrient (N, P, Fe), pharmaceutical (caffeine, diphenhydramine) and nutrient plus pharmaceutical additions in montane and urban streams in three catchments in northern Utah, United States. We included N, P, and Fe as these nutrients may limit biofilm growth and activity at their natural levels (Van Horn et al., 2011; Larson et al., 2018) and human activities have dramatically increased N and P levels in streams worldwide (Seitzinger et al., 2010). We included caffeine and diphenhydramine in our pharmaceutical addition treatment as both are widespread contaminants in human-impacted streams (Kolpin et al., 2002) and have previously been shown to influence biofilm growth, function, and composition (Lawrence et al., 2005; Rosi-Marshall et al., 2013). We incorporated a nutrient plus pharmaceutical addition treatment to examine the combined effects of the two contaminant classes which may enter streams through similar sources (i.e., wastewater effluent, livestock) and thus co-occur (Rosi-Marshall and Royer, 2012). We identified and compared bacterial core communities (core) for each contaminant treatment by land-use combination, where bacterial core taxa were defined as all taxa that occurred in at least 75% of the samples in a specific grouping. We measured ambient nutrient and pharmaceutical concentrations to examine the potential for existing pollution to influence bacterial responses to experimental nutrient and pharmaceutical additions. We hypothesized that nutrients would select for bacterial cores dominated by copiotrophic taxa, while pharmaceuticals would select for cores composed of contaminant-degrading taxa. Further, we hypothesized that existing nutrient and pharmaceutical pollution in urban streams would decrease the sensitivity of biofilm bacteria to experimental additions of these pollutants.

## Materials and Methods

### Study Areas

We conducted nutrient and pharmaceutical addition bioassays at sites located along a gradient of montane to urban land-use in three catchments, Logan River, Red Butte Creek, and Middle Provo River, located along the Wasatch Range in northern Utah, United States. These catchments contain different mixtures of upland forest/sage-steppe, urban, sub-urban, and agricultural land-use. The Logan River originates in the Bear River Range, passes through a series of three hydroelectric dams located near the base of the mountains, and then flows through a mixture of urban and agricultural land-uses. Red Butte Creek flows from the foothills of the Wasatch Range where it passes through a water supply reservoir and then makes a rapid transition into the built-out, urban environment of the University of Utah campus and Salt Lake City. Located between the Jordanelle and Deer Creek Reservoirs, the Middle Provo River flows through the Heber Valley, an area undergoing a rapid change from rural to urban land-use. Our research was conducted during the summer of 2015.

### Stream Biofilm Contaminant Exposure Substrates

We examined the effects of nutrients and pharmaceuticals on stream biofilm bacterial community composition using CES (photograph provided in Supplementary Figure 1; Rosi-Marshall et al., 2013; Costello et al., 2016). To construct CES, we filled 30-mL plastic cups (Polycon, Madan Plastics) with agar amended with one of four contaminant treatments: no additions (control), 0.5M N + 0.5M P + 0.5 mM Fe [nutrient (Nu) addition; 7000 mg N L^–1^ + 15500 mg P L^–1^ + 28 mg Fe L^–1^], 2.5 mM caffeine + 2.5 mM diphenhydramine [pharmaceutical (Ph) addition; 490 mg caffeine L^–1^ + 640 mg diphenhydramine L^–1^], and 0.5M N + 0.5M phosphorus + 0.5 mM Fe + 2.5 mM caffeine + 2.5 mM diphenhydramine [combined nutrient and pharmaceutical (NuPh) addition]. Ethylenediaminetetraacetic acid was added in all treatments with Fe. We chose these contaminant treatment concentrations to match N and P concentrations typically used in NDS studies (Tank et al., 2007; Capps et al., 2011). Similarly, previous CES studies have employed caffeine and diphenhydramine concentrations between 2.5 mM (Shaw et al., 2015) and 13–15 mM (Rosi-Marshall et al., 2013). We capped the agar with a porous glass disk and incubated three replicates of each contaminant treatment *in situ* at montane and urban sites for 18–26 days in the Logan River, Red Butte Creek, and Middle Provo River catchments (4 contaminant treatments × 2 land-use types × 3 catchments × 3 replicates = 72). During the in-stream incubation, biofilms which colonized the porous disks capping the CES were exposed to contaminants diffusing out of the agar, which likely declined over time (Costello et al., 2016). In a laboratory study of NDS containing 0.5M N or 0.5M P, initial N and P release rates were near 1 mg h^–1^ and declined log-linearly to 0.1 mg h^–1^ after 21 days (Rugenski et al., 2008). Diphenhydramine and caffeine release rates likely exceeded environmental concentrations by approximately four orders of magnitude. A laboratory experiment of CES containing pharmaceuticals at 2.5 mM concentrations measured average release rates of 49 ng diphenhydramine min^–1^ cm^–2^ and 41 ng caffeine min^–1^ cm^–2^ over 21 days (Shaw et al., 2015). In comparison, a typical wastewater-impacted stream with diphenhydramine concentrations of 100 ng L^–1^ would expose biofilms to 0.002 ng diphenhydramine min^–1^ cm^–2^ (Costello et al., 2016).

At the end of the in-stream incubation, we collected the biofilm-colonized disks in sterile plastic bags (Whirl-Pak^®^) and transported them on ice to the laboratory where they were stored at 4°C. Due to the loss of several CES that occurred during the in-stream incubations, only two replicates of the control and nutrient plus pharmaceutical addition treatments were recovered at the urban site in the Logan River catchment and all bacterial community inferences were based on 70 samples.

### Effects on Bacterial Community Composition

We performed a 16S rRNA gene metabarcoding analysis to examine bacterial community composition in montane and urban stream biofilms exposed to contaminant treatments. We extracted genomic DNA from biofilms using the DNeasy Powersoil Kit (Qiagen, Inc., Germantown, MD, United States). We then targeted the V4 hypervariable region of the 16S rRNA gene using primers 16Sf (5′-GTGCCAGCMGCCGCGGTAA-3′) and 16Sr (5′-GGACTACHVGGGTWTCTAAT-3′). The primers contained a series of repeating 8-bp barcodes, a forward or reverse Illumina primer, linker region, and primer pad to facilitate a dual-indexed Illumina sequencing approach (Caporaso et al., 2012; Kozich et al., 2013; Aanderud et al., 2018). The Invitrogen^TM^ AccuPrime^TM^ Pfx SuperMix was used for the generation of 16S rRNA gene amplicons. The thermocycler settings were: an initial denaturation step of 94°C for 3 min, followed by 35 cycles of denaturation at 94°C for 45 s, annealing at 55°C for 60 s, and elongation at 72°C for 90 s. A final elongation step was set for 72°C for 10 min and samples were then held at 4°C. Samples were then normalized using the SequalPrep^TM^ Normalization Plate (96) Kit (Invitrogen, Carlsbad, CA, United States) and quantified on a Qubit^®^ 2.0 Fluorometer (Thermo Fisher Scientific, Waltham, MA, United States). The samples were sequenced on an Illumina HiSeq 2500 platform (2 × 250; Illumina Biotechnology, San Diego, CA, United States) at the Brigham Young University DNA Sequencing Center^1^. Primer dimers were removed via Pippin Prep (Sage Science, Beverly, MA, United States). Illumina sequence reads were analyzed in *mothur* (v. 1.35.1; Schloss et al., 2009) following the MiSeq SOP protocol (Kozich et al., 2013). We removed chimeras with UCHIME (Edgar et al., 2011) and eliminated chloroplast, mitochondrial, archaeal, and eukaryotic gene sequences based on reference sequences from the Ribosomal Database Project (Cole et al., 2009). We then aligned sequences against the SILVA 128 database (silva.nr_v128.align; Pruesse et al., 2007) with the SEED aligner and created operational taxonomic units (OTUs) based on uncorrected pairwise distances using a minimum coverage of 99% and minimum pairwise sequence similarity of 97%. We used the ‘phyloseq’ package (McMurdie and Holmes, 2013) in R (R Core Team, 2017) to combine taxonomy files and OTU tables generated in *mothur*. Samples contained a total of 1,856,706 sequences and 16,438 unique OTUs with a mean sequencing coverage of 98 ± 0.1%. We rarefied data to the smallest sample size (27,704 sequences, 18,984 unique OTUs after rarefication) and then assigned OTUs from the total bacterial community into two abundance categories: abundant (≥0.1% relative abundance) and rare (<0.1% relative abundance; Pedrós-Alió, 2012; Aanderud et al., 2015). The resulting rarefied dataset was used to perform all of the following analyses.

### Nutrient and Pharmaceutical Effects on Biofilm Bacterial Core Communities in Montane and Urban Streams

We identified bacterial core communities (cores) for each contaminant treatment by land-use combination (i.e., control montane, control urban, nutrient montane, etc.) by selecting all OTUs that occurred in at least 75% of the samples in a specific grouping using the ‘microbiome’ package (Lahti et al., 2017) in R. We defined bacterial cores using a 75% persistence cutoff to identify bacterial taxa that consistently responded to contaminant treatments in montane and urban biofilms and thus characterize common bacterial responses to contaminant additions at each type of land-use. Our 75% persistence cutoff also ensured sufficient number of taxa above the cutoff threshold in all bacterial cores (>200 OTUs). We visualized the composition of bacterial cores with Krona plots (Ondov et al., 2011) generated in R. Krona plots displayed the percentages of bacterial core taxa, calculated as a taxon’s total sequence count divided by the total sequence count of all taxa across all samples contributing to a bacterial core. We employed the same method to calculate and display the contribution of abundant compared with rare OTUs.

We examined the effects of land-use, nutrient addition, and pharmaceutical addition on the composition of bacterial cores. We visualized community differences among cores by performing a principal coordinate analysis using a Bray–Curtis abundance-based distance matrix from the relative recovery of OTUs with the *vegdist* function in the ‘vegan’ package (Oksanen et al., 2014). We then performed a PERMANOVA analysis (Anderson, 2001) using the *adonis* function to test for the effects of land-use, nutrient addition, and pharmaceutical addition with catchment nested within land-use, nutrient addition, and pharmaceutical addition.

Next, we focused on the effects of nutrients and pharmaceuticals. We calculated bacterial core diversity as Shannon’s diversity index, and richness as the number of OTUs in each bacterial core. We performed separate two-way ANOVAs for bacterial cores at montane and urban sites, where nutrient addition and pharmaceutical addition were included as fixed effects with catchment nested within nutrient addition and pharmaceutical addition. Treatment differences were compared using Tukey’s *post hoc* tests. Last, to visualize the distinctiveness of bacterial cores exposed to contaminant treatments at each type of land-use, we generated area-proportional Euler diagrams based on counts of the number of shared and unique taxa in bacterial cores of each contaminant treatment at each type of land-use using the ‘eulerr’ package (Larsson, 2019). We visualized the composition of unique taxa in bacterial cores with Krona plots created using the methods described previously.

### Effects on Biofilm Biomass

We characterized the effects of nutrients and pharmaceuticals on stream biofilm biomass to assess contaminant effects on biofilm growth and facilitate comparisons to existing CES studies where biomass is frequently measured. We measured ash-free dry mass (AFDM) and chlorophyll *a* concentrations of biofilms grown on CES to estimate total organic biomass and photoautotrophic biomass, respectively. One-half of each fritted glass disk was used for chlorophyll determination and the other for AFDM. The disk area was estimated with the Image J software (Schneider et al., 2012). Chlorophyll was extracted in hot ethanol, refrigerated overnight, and measured on a Turner Designs TD-700 Fluorometer with pheophytin correction after acidification (Hauer and Lamberti, 2011). AFDM samples were dried for 2 days at 60°C, combusted at 450°C for 2 h, and the mass lost during combustion used to calculate AFDM (APHA, 1998). We examined the effects of nutrient addition and pharmaceutical addition on chlorophyll and AFDM in montane and urban streams using two-way ANOVAs as described above for core comparisons.

### Ambient Nutrient and Pharmaceutical Concentrations

We measured ambient nutrient concentrations to examine the potential for existing nutrient pollution to influence biofilm bacteria. We collected grab samples for total nitrogen (TN) and total phosphorus (TP) analyses, samples of stream water filtered through pre-combusted Whatman GF/F filters for nitrite + nitrate (hereafter ${}^{{\text{NO}}_{3}^{-}}$), ammonium (${}^{{\text{NH}}_{4}^{+}}$), and soluble reactive phosphorus (SRP) analyses, and 1.5-mL samples of stream water filtered through a 0.2-μm filter for dissolved total Fe and dissolved ferrous Fe analyses. We immediately added 40-μl of Ferrozine to the dissolved ferrous Fe samples. All samples were collected in duplicate. TN was quantified using a potassium persulfate digestion (Nydahl, 1978) followed by a cadmium reduction for ${}^{{\text{NO}}_{3}^{-}}$ (APHA, 1998, EPA method 353.2). TP was quantified using a potassium persulfate digestion followed by an ascorbic acid molybdenum reaction for SRP (Murphy and Riley, 1962, EPA method 365.1). ${\text{NO}}_{3}^{-}$ and SRP were measured using the methods described above without digestion. ${}^{{\text{NH}}_{4}^{+}}$ was quantified with an automated alkaline phenolhypochlorite reaction followed by spectrophotometric analysis (EPA method 350.1, Solorzano, 1969; APHA, 1998). Dissolved total Fe and dissolved ferrous Fe were measured colorimetrically at a wavelength of 562 nm (Stookey, 1970). The method detection limits were 8.0 μg L^–1^ for TN, 12.0 μg L^–1^ for TP, 6.0 μg L^–1^ for ${}^{{\text{NH}}_{4}^{+}}$, 4.0 μg L^–1^ for ${}^{{\text{NO}}_{3}^{-}}$, 1.0 μg L^–1^ for SRP, and 17.1 μg L^–1^ for dissolved total Fe and dissolved ferrous Fe. We performed one-way ANOVAs with land-use as a fixed effect and catchment nested within land-use to test for differences in nutrients concentrations between montane and urban streams.

To examine the potential for pharmaceutical pollution to influence biofilm bacteria, we measured ambient concentrations of pharmaceuticals by deploying Passive Organic Chemical Integrative Samplers (POCIS) in streams at the urban site in each catchment (Alvarez et al., 2004). Pharmaceutical POCIS containing Waters Oasis HLB^TM^ polymer sorbent were obtained from Environmental Sampling Technologies (EST, Inc., St. Joseph, MO, United States) and deployed at each location in a stainless-steel strainer (Zicome Collapsible Vegetable Steamer 7.5 × 7.3 × 2.3 inches) except at one location where a protective covering was fashioned out of plastic landscape cloth. POCIS are typically deployed in the field for an average of 30 days, although deployments have ranged from weeks to months (Alvarez, 2010). Accordingly, we deployed our POCIS for 20–26 days during the summer and fall of 2015. The concentrations of 19 pharmaceuticals were measured with high performance liquid chromatography combined with tandem mass spectrometry (Method 1694; EPA, 2007) by the University of Nebraska Water Sciences Laboratory.

## Results

### Bacterial Core Communities

**FIGURE 1:**
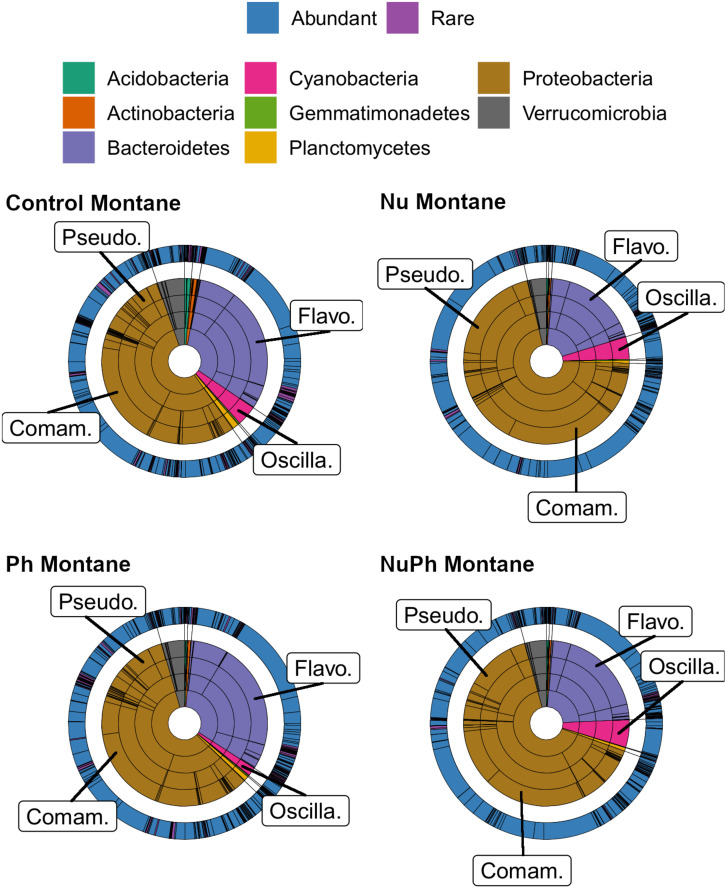
Krona plots of biofilm bacterial cores in control, nutrient (Nu), pharmaceutical (Ph), and nutrient plus pharmaceutical (NuPh) treatments in montane streams. Bacterial cores defined as OTUs present in more than 75% of samples of a given contaminant treatment in montane streams across the three catchments. Inner rings, in order from smallest to largest, show percentages of individual taxa at phylum, class, order, and family levels and colors indicate different phyla. The outer ring shows the percentage of individual taxa at the OTU level and colors indicate abundant (≥0.1% relative abundance of total bacterial community) and rare OTUs (<0.1% relative abundance of total bacterial community). To simplify Krona plots, we only included taxa belonging to eight most abundant phyla across all bacterial cores, which comprised at least 99% of the abundance of bacterial cores at montane sites. Bacterial cores based on 16S rRNA gene community libraries (97% similarity cut-off). Top families are labeled with the following abbreviations: Comam. = Comamonadaceae, Flavo. = Flavobacteriaceae, Oscilla. = Oscillatoriales, and Pseudo. = Pseudomonadaceae.

**FIGURE 2:**
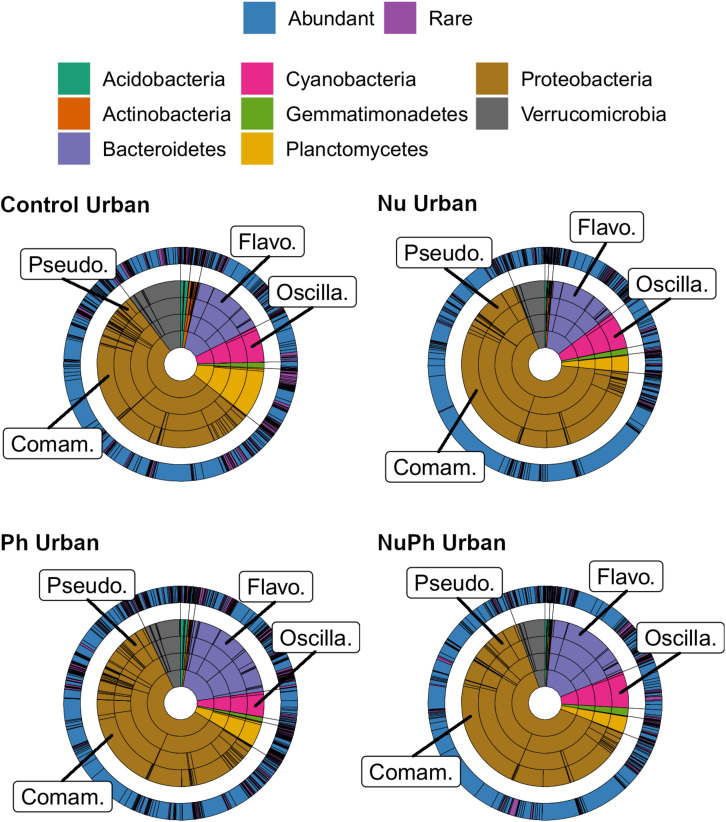
Krona plots of biofilm bacterial cores on control, nutrient (Nu), pharmaceutical (Ph), and nutrient plus pharmaceutical (NuPh) treatments at urban sites. Krona plots are described in Figure 1. The eight phyla comprised at least 99% of the abundance of bacterial cores at urban sites. Top families are labeled with the following abbreviations: Comam. = Comamonadaceae, Flavo. = Flavobacteriaceae, Oscilla. = Oscillatoriales, and Pseudo. = Pseudomonadaceae.

**FIGURE 3:**
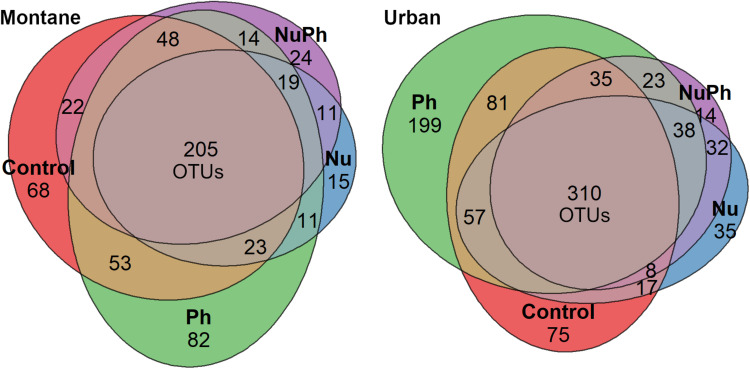
Venn diagrams of shared and unique bacterial core OTUs in control, nutrient (Nu), pharmaceutical (Ph), and nutrient plus pharmaceutical (NuPh) treatments in montane (left diagram) and urban streams (right diagram). Values are the number of OTUs in each section and section areas are proportional to the number of OTUs. Bacterial cores in montane streams contained a total of 611 OTUs and bacterial cores in urban streams contained a total of 959 OTUs.

Overall, biofilm bacterial cores were composed of mostly a few abundant taxa (Figures 1, 2). Bacterial cores at montane and urban sites contained a mean (± SEM) of 385 ± 37 and 576 ± 73 of OTUs, which represented only 2 and 3% of OTUs in the total bacterial community, respectively (Supplementary Table 1). Abundant taxa comprised 34 ± 3.4 and 24 ± 2.4% of bacterial core OTUs at montane and urban sites, respectively (Supplementary Table 1). In comparison, abundant taxa comprised only 0.81% of OTUs in the total bacterial community. The most abundant bacterial core phyla included Proteobacteria (mean relative abundance ± SEM: montane = 62 ± 3.8%; urban = 60 ± 2.9%), Bacteroidetes (montane = 27 ± 3.2%; urban = 16 ± 1.5%), Verrucomicrobia (montane = 4.4 ± 0.34%; urban = 7.2 ± 1.10%), and Cyanobacteria (montane = 3.9 ± 0.75%; urban = 6.5 ± 0.54%; Figures 1, 2). Control, nutrient, pharmaceutical, and nutrient plus pharmaceutical bacterial cores shared 35 and 32% of their OTUs at montane and urban sites, respectively (Figure 3).

### Land-Use and Contaminant Effects on Bacterial Core Structure

**FIGURE 4:**
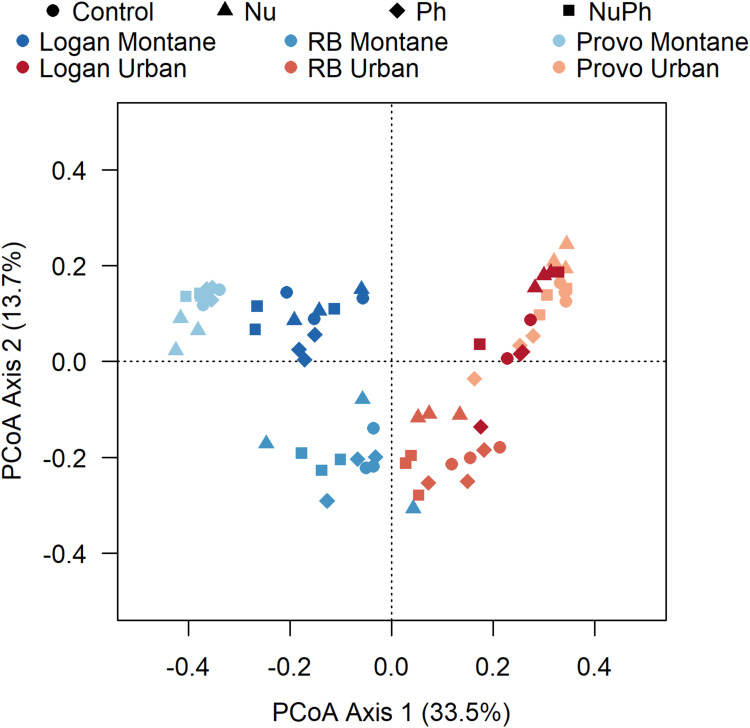
Principle coordinate analysis (PCoA) generated using Bray–Curtis distances on a sample × OTU matrix of 16S rRNA gene community libraries. Bacterial cores in control, nutrient addition, pharmaceutical addition, and nutrient plus pharmaceutical addition treatments are represented as different shapes with montane and urban streams in the Logan, Red Butte (RB), and Middle Provo (Provo) catchments represented as different colors.

Bacterial cores were primarily structured by urban and montane land-use and secondarily by nutrient and pharmaceutical additions. In the PCoA ordination, the two different land-uses clearly separated bacterial cores along the primary axis (Figure 4). In general, bacterial cores at montane sites in the three catchments were compositionally distinct, while bacterial cores at urban sites were more similar to each other. PERMANOVA analysis confirmed the separation in ordination space, with land-use explaining 27% of the variation (land-use: *F*_1_,_66_ = 27, *p* < 0.001, *R*^2^ = 0.27). In comparison to land-use, nutrients and pharmaceutical additions had less of an impact but still helped separate bacterial cores along axis 2 and contributed to the formation of distinct bacterial cores based on PERMANOVA results (Nu: *F*_1_,_66_ = 5.9, *p* < 0.001, *R*^2^ = 0.06, Ph: *F*_1_,_66_ = 2.4, *p* = 0.02, *R*^2^ = 0.02).

### Nutrient Effects on Bacterial Cores

**FIGURE 5:**
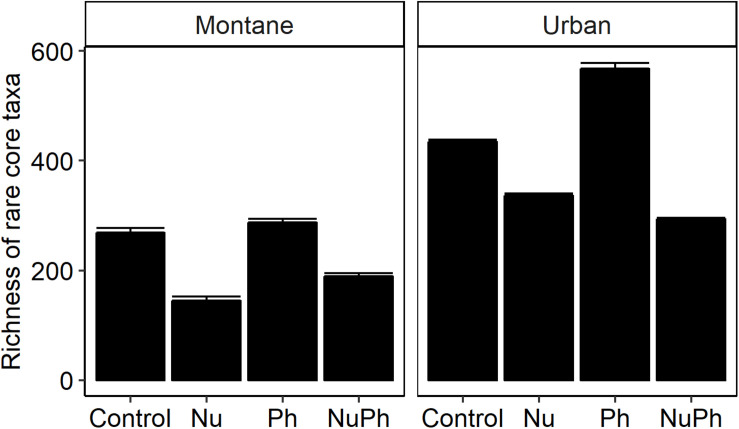
Richness of bacterial cores in control, nutrient addition, pharmaceutical addition, and nutrient plus pharmaceutical addition treatments in montane (left panel) and urban streams (right panel). Values are means (*n* = 8–9) shown with standard error of the mean. In two-way ANOVAs, nutrients and pharmaceuticals had interactive effects on bacterial core richness, with nutrients decreasing and pharmaceuticals increasing richness in montane (Nu addition × Ph addition, *F*_1_,_24_ = 11.7, *p* = 0.002; Nu addition, *F*_1_,_24_ = 677, *p* < 0.001; Ph addition, *F*_1_,_24_ = 70.6, *p* < 0.001) and urban streams (Nu addition × Ph addition, *F*_1_,_22_ = 188, *p* < 0.001; Nu addition, *F*_1_,_22_ = 846, *p* < 0.001; Ph addition, *F*_1_,_22_ = 64.7, *p* < 0.001). All treatments were significantly different within each type of land-use.

Nutrients enhanced the dominance of the Pseudomonadaceae and Comamonadaceae in both nutrient and nutrient plus pharmaceutical bacterial cores (Figures 1, 2). The abundance of the Pseudomonadaceae, a family containing seven core taxa, was at least 5- and 3-fold higher in nutrient and nutrient plus pharmaceutical cores, respectively, than in control cores (Figures 1, 2). Comamonadaceae, which contained the highest abundance of OTUs in control bacterial cores and contained 44 core taxa, was 1.7- and 1.5-more abundant in nutrient and nutrient plus pharmaceutical cores as compared to controls at urban sites, respectively (Figure 1). Nutrients depressed bacterial core richness by at least 19 and 26% at montane and urban sites, respectively (Figure 5).

### Pharmaceutical Effects on Bacterial Cores

Pharmaceutical effects generally contrasted with those of nutrients, as pharmaceuticals increased bacterial core richness (Figure 5) but had few effects on abundant families (Figures 1, 2). Pharmaceuticals had the most dramatic effect on richness in urban streams, where pharmaceutical exposure increased bacterial core richness 38% above controls (Figure 5B). In montane streams, pharmaceuticals only increased bacterial core richness by 6% above controls (Figure 5A). Pharmaceuticals had a minimal effect on abundant families with the exception of Pseudomonadaceae, which was 4.8- and 1.5-times more abundant in pharmaceutical as compared to control bacterial cores at urban and montane sites, respectively (Figures 1, 2).

**FIGURE 6:**
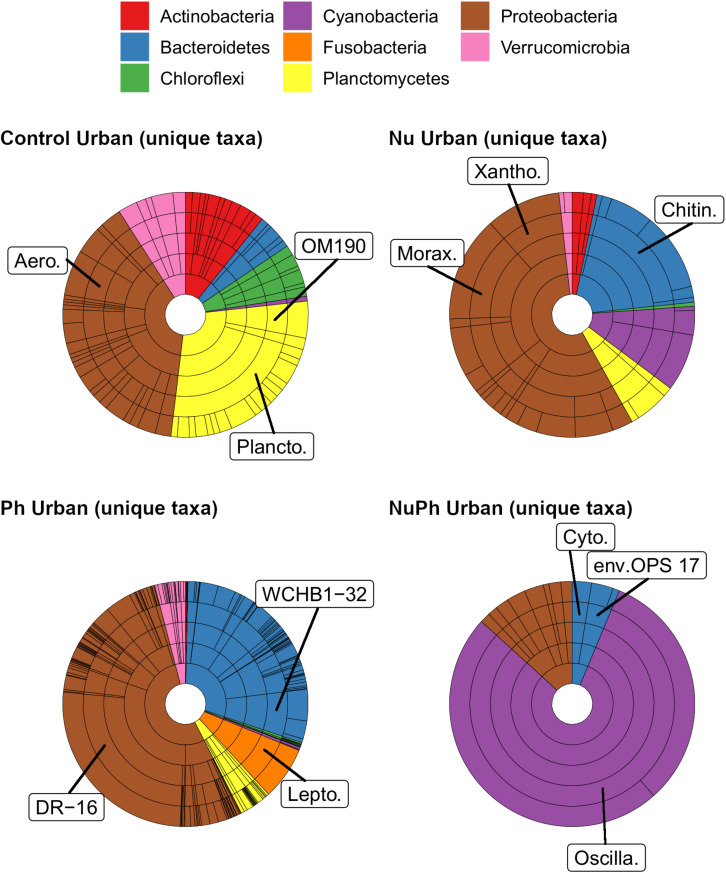
Krona plots of unique taxa in bacterial cores in control, nutrient (Nu), pharmaceutical (Ph), and nutrient plus pharmaceutical (NuPh) treatments in urban streams. Rings, in order from smallest to largest, show percentages of individual taxa at phylum, class, order, family, and OTU levels. Colors indicate different phyla. Note that the phyla and color key are different from those in Figures 1, 2. To simplify Krona plots, we only included taxa belonging to eight most abundant phyla of unique taxa, which comprised at least 96% of the abundance of unique taxa. Top families of unique taxa in each bacterial core are labeled using the following abbreviations: Aero. = Aeromonadaceae, Chitin. = Chitinophagaceae, Cyto. = Cytophagaceae, DR-16 = DR-16 family, env.OPS 17 = Sphingobacteriales env.OPS 17, Lepto. = Leptotrichiaceae, Morax. = Moraxellaceae, OM190 = OM190 family, Oscilla. = Oscillatoriales, Plancto. = Planctomycetaceae, WCHB1-32 = WCHB1-32 family, Xantho. = Xanthomonadaceae.

Pharmaceutical bacterial cores were distinct, containing many unique taxa associated with contaminant-degradation. Based on the Venn diagrams of the OTUs found in contaminant treatment bacterial cores at urban sites, 21% of OTUs were unique to the pharmaceutical bacterial cores, while only 7.8, 3.7, and 1.5% were unique to the control, nutrient, and nutrient plus pharmaceutical bacterial cores, respectively (Figure 3B). The pharmaceutical bacterial core was less unique at montane sites, where 14, 11, 2.5, and 4.0% of OTUs were unique to pharmaceutical, control, nutrient, and nutrient plus pharmaceutical bacterial cores, respectively (Figure 3A). Top families of unique pharmaceutical bacterial core taxa at urban sites (Figure 6) included DR-16 (Betaproteobacteria; 25% of unique pharmaceutical bacterial core OTUs), WCHB1-32 (Bacteroidetes; 6.8%), and Leptotrichiaceae (Fusobacteria; 6.8%). At montane sites, top families of unique pharmaceutical bacterial core taxa included unclassified Bacteroidetes (10.0%), Oscillatoriales (9.8%), and Sphingobacteriales env.ops 17 (Bacteroidetes; 6.9%).

### Nutrient Plus Pharmaceutical Effects on Bacterial Cores

Although the composition of nutrient plus pharmaceutical bacterial cores closely resembled that of nutrient bacterial cores, nutrient plus pharmaceutical bacterial cores also contained taxa not found in other contaminant treatment bacterial cores and the unique taxa in the nutrient plus pharmaceutical core were distinct from unique taxa in the control, nutrient, and pharmaceutical cores at urban sites (Figure 6). Oscillatoriales comprised 80 and 11% of the OTUs unique to nutrient plus pharmaceutical bacterial cores at urban and montane sites, respectively (Figure 6). Other major families of unique nutrient plus pharmaceutical bacterial core taxa at urban sites included Sphingobacteriales env.OPS 17 (Bacteroidetes; 3.7% of unique nutrient plus pharmaceutical bacterial core OTUs), Cytophagaceae (Bacteroidetes; 2.5%), and Nitrosomonadaceae (Betaproteobacteria; 2.5%), while major families at montane sites included Verrucomicrobiaceae (Verrucomicrobia; 19%), Rhodobacteraceae (Alphaproteobacteria; 17%), and unclassified Burkholderiales (Betaproteobacteria; 6.6%).

### Ambient Nutrients and Pharmaceuticals

Urban biofilms were exposed to distinct signatures of nutrient and pharmaceutical pollution associated with the different types of land-use in the catchment. Although variable, ambient nutrient concentrations were generally higher in urban compared to montane streams (Table 1): dissolved and total N and P and total dissolved Fe concentrations were significantly greater in urban as compared to montane streams (all models one-way ANOVAs with land-use as the predictor variable: *p* < 0.02). The increase in ambient nutrient concentrations was likely due to human land-use, as differences in the form and ratio of N and P across the three catchments reflected differences in the intensity of urbanization in the three watersheds. The urban stream in the Logan catchment, which is impacted by a mixture of agricultural and urban land-use, had greater ${}^{{\text{NO}}_{3}^{-}}$ concentrations and DIN:SRP ratios than urban streams in the Red Butte and Middle Provo catchments, potentially reflecting chemical fertilizer inputs associated with agriculture (Kaushal et al., 2011). In contrast, P concentrations increased between montane and urban streams in Red Butte Creek and Middle Provo, potentially reflecting the influence of urban land-use, which generally increases P inputs due to sewage and septic inputs (Paul and Meyer, 2001; Walsh et al., 2005; Kaushal et al., 2011). In addition, the Middle Provo urban stream is also the only stream in our study located downstream of a wastewater treatment plant and the increase in P concentrations between montane and urban streams in this catchment may reflect the influence of treated wastewater inputs.

**TABLE 1:** Ambient nutrient concentrations in montane and urban streams in the Logan River, Red Butte Creek, and Middle Provo River catchments during 2015 CES bioassays.

	Logan River		Red Butte Creek		Middle Provo River	
	Montane	Urban	Montane	Urban	Montane	Urban
TN (mg L^–1^)	0.22 (0.032)	0.41 (0.008)	0.10 (0.015)	0.31 (0.015)	0.19 (0.002)	0.31 (0.002)
TP (mg L^–1^)	0.02 (0.004)	0.02 (0.004)	0.03 (0.008)	0.08 (0.001)	0.01 (<0.001)	0.03 (<0.001)
${}^{{\text{NH}}_{4}^{+}}$ (mg L^–1^)	< 0.01 (0.002)	0.01 (0.001)	0.01 (0.001)	0.01 (<0.001)	0.01 (<0.001)	0.01 (0.002)
${}^{{\text{NO}}_{3}^{-}}$ (mg L^–1^)	0.14 (0.002)	0.28 (0.001)	0.04 (<0.001)	0.10 (0.002)	0.05 (<0.001)	0.11 (0.002)
SRP (mg L^–1^)	<0.01 (<0.001)	<0.01 (<0.001)	0.01 (<0.001)	0.01 (<0.001)	<0.01 (0.002)	0.01 (<0.001)
Molar TN:TP	21.8 (0.87)	49.4 (10.10)	8.3 (1.16)	8.80 (0.34)	70.5 (0.57)	21.7 (<0.01)
Molar DIN:SRP	112 (9.09)	186 (18.3)	19.8 (1.72)	23.0 (0.25)	45.1 (23.12)	31.9 (2.02)
Dissolved total Fe (μg L^–1^)	8.57 (<0.001)	16.3 (7.76)	8.57 (<0.001)	31.9 (2.13)	41.1 (2.84)	12 (9.59)
Dissolved ferrous Fe (μg L^–1^)	8.57 (<0.001)	8.57 (<0.001)	8.57 (<0.001)	20.23 (11.7)	8.57 (<0.001)	8.57 (<0.001)

*Chemistry values are means on date of CES deployment (*n* = 2) followed by standard error of the mean in parentheses. TN, total nitrogen, TP, total phosphorus, $N\,H_{4}^{+}$, ammonium, $N\,O_{3}^{-}$, nitrate, SRP, soluble reactive phosphorus, DIN, molar dissolved inorganic nitrogen. One half of the method detection limit was used to calculate means and standard errors of nutrient concentrations when values were below the method detection limit (TN = 0.008 mg L^–1^, TP = 0.012 mg L^–1^, $N\,H_{4}^{+}$ = 0.006 mg L^–1^, $N\,O_{3}^{-}$ = 0.004 mg L^–1^, SRP = 0.001 mg L^–1^, dissolved total and dissolved ferrous Fe = 17.13 μg L^–1^).*

Similarly, urban biofilms were also exposed to different forms of pharmaceutical pollution associated with human land-use in the three catchments (Table 2). Caffeine, one of the contaminants in our pharmaceutical treatment, and 1,7-dimethylzanthine, a metabolite of caffeine, were present in urban streams in all three catchments. Diphenhydramine, the other contaminant in our pharmaceutical treatment, was only detected in the Logan River and Red Butte Creek catchments. The different types of human land-use created distinct chemical signatures of human- and livestock-associated pharmaceuticals. Sulfa antibiotics (sulfamethazine and sulfamethoxazole), pharmaceuticals primarily associated with livestock (Mathew et al., 2007), were most frequently detected in the Logan and Middle Provo catchments, which are both impacted by agricultural land-use. Pharmaceuticals associated with human consumption, including 1,7-dimethylxanthine (a metabolite of caffeine), acetaminophen, caffeine, carbamazepine, and cotinine (a metabolite of nicotine) were detected in all three catchments, reflecting anthropogenic impacts found in both rural and urban environments.

**TABLE 2:** Concentrations of pharmaceuticals that accumulated in Polar Organic Chemical Integrated Samplers (POCIS) in urban streams in the Logan River, Red Butte Creek, Middle Provo River catchments during summer (June–July) and fall [September (Sept.)–October (Oct.)].

	Logan river		Red butte creek		Middle provo river	
Compound	June 18–July 14	Sept. 13–Oct. 10	June 5–June 25	Sept. 14–Oct. 10	June 12–July 6	Sept. 27–Oct. 23
1,7-Dimethylxanthine	BDL	0.09	0.05	0.10	0.05	0.05
Acetaminophen	BDL	0.03	0.06	0.09	0.05	0.06
Amphetamine	0.03	0.10	BDL	0.35	BDL	BDL
Azithromycin	BDL	BDL	BDL	BDL	BDL	BDL
Caffeine	1.52	1.51	1.98	4.89	0.45	0.55
Carbamazepine	0.04	0.03	0.03	BDL	0.12	0.08
Cimetidine	BDL	BDL	BDL	BDL	BDL	BDL
Cotinine	0.08	0.06	0.08	0.21	0.03	0.05
Diphenhydramine	0.04	0.01	0.01	BDL	BDL	BDL
MDA	BDL	BDL	BDL	BDL	BDL	BDL
MDMA	0.01	<0.01	BDL	BDL	BDL	BDL
Methamphetamine	0.02	0.01	0.32	0.58	BDL	BDL
Morphine	BDL	BDL	BDL	BDL	BDL	BDL
Phenazone	BDL	BDL	BDL	BDL	BDL	BDL
Sulfachloropyridazine	BDL	BDL	BDL	BDL	BDL	BDL
Sulfamethazine	0.58	0.58	BDL	BDL	0.65	0.17
Sulfamethoxazole	BDL	0.28	BDL	0.04	0.05	0.05
Thiabendazole	BDL	BDL	BDL	BDL	BDL	BDL

*Pharmaceutical concentrations are expressed as ng POCIS^–1^. Dates in 2015 when POCIS were deployed at each site and concentrations of pharmaceuticals that accumulated in POCIS (ng POCIS^–1^) are reported. Method detection limit is 0.002 ng for all pharmaceuticals. BDL, below detection limit; MDA, 3,4-methylenedioxy-amphetamine; MDMA, 3,4-methylenedioxy-methamphetamine.*

### Contaminant Effects on Biofilm Biomass

**FIGURE 7:**
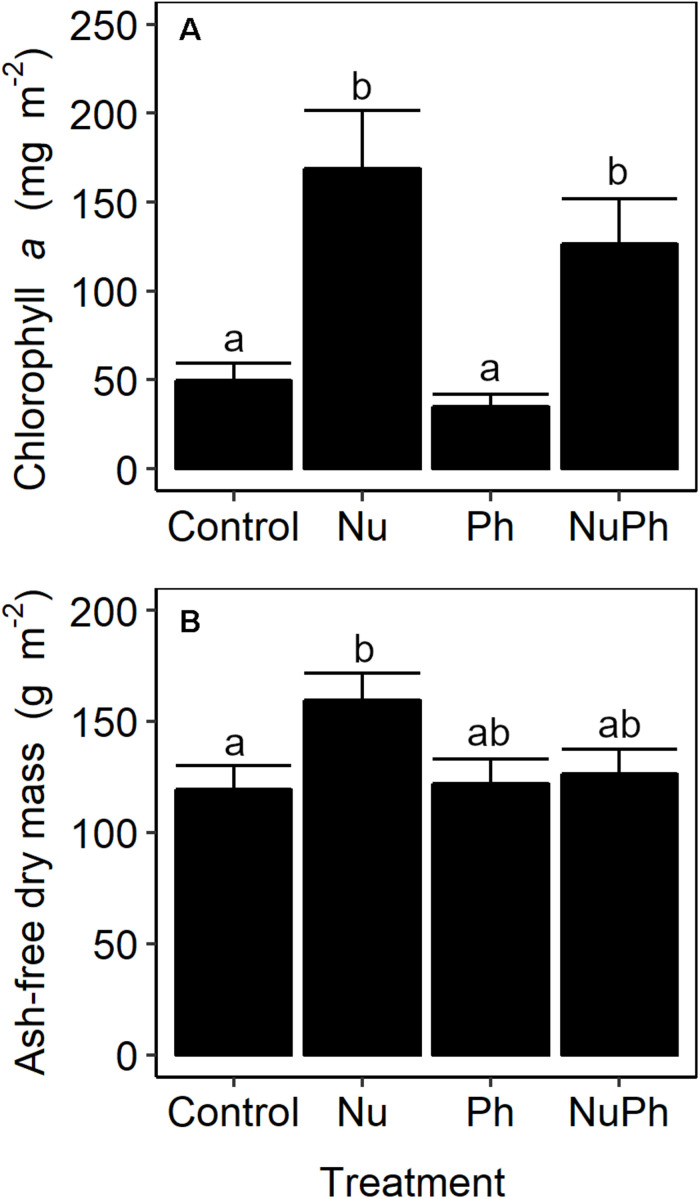
Chlorophyll *a* concentrations **(A)** and ash-free dry mass **(B)** of biofilms grown on control, nutrient addition, pharmaceutical addition, and nutrient plus pharmaceutical addition treatments in urban streams. Values are means ± SEM (*n* = 3). Treatments with different lowercase letters were significantly different from each other (all *p* ≤ 0.03).

Urban biofilm biomass was nutrient-limited. Nutrient additions increased chlorophyll *a* concentrations by approximately 250% (Figure 7A; Nu: *F*_1_,_47_ = 36, *p* < 0.001), but increased AFDM by only 25% (Figure 7B; Nu: *F*_1_,_49_ = 5.2, *p* = 0.03). Pharmaceuticals in urban streams and nutrients and pharmaceuticals in montane streams did not influence biofilm biomass.

## Discussion

Nutrient and pharmaceutical additions created distinct bacterial cores in stream biofilms at montane and urban sites across the three catchments. Nutrients enhanced the dominance of Pseudomonadaceae and Comamonadaceae in bacterial cores and depressed bacterial core richness. Pseudomonadaceae and Comamonadaceae both contain many opportunistic copiotrophs often dominating nutrient-rich environments (Pinhassi and Berman, 2003; Yu et al., 2017; Jani et al., 2018; Roberto et al., 2018) and are members of Gammaproteobacteria and Betaproteobacteria, respectively, both copiotrophic Proteobacterial classes (Shi et al., 1999; Fierer et al., 2007a). Additions of N, P, and Fe, the nutrients included in our treatment, likely led to the dominance of these copiotrophs, which were able to outcompete other taxa for these nutrients, ultimately reducing richness (Mittelbach et al., 2001; Besemer, 2015). Nutrient pollution has previously demonstrated a negative effect on bacterial richness within a nutrient-rich urban stream, where nitrate concentrations were four-times greater than pristine streams and biofilm bacterial richness and evenness estimates were visibly lower and under the dominance of several species (Lear et al., 2009). While the results of our CES experiment suggest nutrient enrichment may depress bacterial core richness, the composition of biofilms grown on CES likely differed from natural biofilms at our study sites. A previous CES study which employed cellulose sponges a biofilm colonization substrates found that bacterial and fungal diversity were on the low end of value reported for stream leaf litter and sediments, potentially due to the structural homogeneity of the CES substrate, short incubation time, and/or microbial identification methodology (Hoellein et al., 2010). Research examining the effects of nutrient enrichment on the bacterial core of natural biofilms is an important avenue of future research. Diminished bacterial richness associated with nutrient additions may have important implications for stream ecosystems, as the loss of bacterial taxa may also reduce genetic diversity and, consequently, biofilm resilience to disturbance (Sogin et al., 2006; Pedrós-Alió, 2012).

The effects of pharmaceuticals largely contrasted those of nutrients, as pharmaceuticals increased bacterial core richness and fostered unique taxa associated with contaminant tolerance and/or degradation. The pharmaceuticals used in our study, caffeine and diphenhydramine, may have served as a C and/or energy source for taxa capable of degrading one or both compounds. Indeed, pharmaceuticals increased the relative abundance of Pseudomonadaceae, a family which contains many taxa known for their ability to degrade toxic organic molecules (Palleroni, 2010) and includes the majority of bacteria capable of degrading caffeine (Summers et al., 2015). Experimental diphenhydramine additions have previously been shown to increase the abundance *Pseudomonas* sp. in stream biofilms (Rosi-Marshall et al., 2013). Further, a small percentage (0.9%) of unique pharmaceutical bacterial core taxa at montane sites belonged to the Anaerolineaceae (Chloroflexi), a family associated with diphenhydramine degradation in anaerobic digester sludge (Wolfson et al., 2018). Many taxa that were unique to pharmaceutical bacterial cores are associated with the breakdown of complex C substrates or are commonly found in highly contaminated environments. DR-16, which contained the highest abundance of unique pharmaceutical bacterial core taxa at urban sites, resides in a quinoline degrading microbial consortium and may help degrade aromatic compounds (Wu et al., 2015). Other highly abundant families of unique pharmaceutical bacterial core taxa at urban sites included WCHB1-32, a family found in a hydrocarbon- and chlorinated-solvent-contaminated aquifer undergoing bioremediation (Dojka et al., 1998) and Leptotrichiaceae, a family containing pathogenic bacteria (Lory, 2014) associated with sewage infrastructure (Shanks et al., 2013; Gonzalez-Martinez et al., 2018) that often contains pharmaceuticals. However, pharmaceutical release rates from CES likely exposed CES biofilms to pharmaceutical concentrations that exceeded environmental conditions by approximately four orders of magnitude (Shaw et al., 2015; Costello et al., 2016), likely resulting in more pronounced pharmaceutical effects. Future CES studies that includes environmentally-relevant pharmaceutical concentrations and dose-response curves (Costello et al., 2016) could provide insight into the ecological effects of pharmaceutical pollution and how these effects change with concentration.

Our results highlight the potential interplay between pharmaceutical pollution, diversity, and pharmaceutical degradation. The increase in bacterial core richness associated with pharmaceutical addition is notable, as contaminant degradation rates may increase with microbial community diversity (Dell’Anno et al., 2012; Hernandez-Raquet et al., 2013; Delgado-Baquerizo et al., 2016). Bacterial biodiversity may provide a rich set of taxa that can act as a consortium supporting specialized metabolic pathways required to degrade complex organic pollutants (Fuentes et al., 2014; Jousset et al., 2017). However, we are only able to hypothesize that bacteria degraded caffeine and diphenhydramine. The pharmaceutical-induced compositional shifts that we found may also result from toxicity and/or tolerance to these contaminants. Stable isotope probing, which tracks the incorporation of an isotopically-labeled substrate of interest into specific taxa within a microbial community (Neufeld et al., 2007), may identify potential pharmaceutical-degrading taxa. Future studies that examine the potential role of biofilm bacterial taxa in pollutant degradation may reveal the importance of this group in stream ecosystem function and resilience to anthropogenic pollution (Jousset et al., 2017).

Nutrients had much stronger effects than pharmaceuticals on bacterial cores when the two contaminant classes were added in combination. The composition of the top bacterial taxa, richness, and diversity in the nutrient plus pharmaceutical bacterial cores largely resembled those in the nutrient bacterial cores. Unexpectedly, nutrient plus pharmaceutical bacterial cores contained taxa not found in nutrient or pharmaceutical bacterial cores. The vast majority of unique nutrient plus pharmaceutical bacterial core taxa in urban streams belonged to the order Oscillatoriales (Cyanobacteria), suggesting the taxa may possess superior competitive abilities under combined nutrient and pharmaceutical enriched conditions. Indeed, Oscillatoriales outcompeted other bacteria for energy and nutrients under N-rich conditions in salt marsh communities (Kearns et al., 2016) and increased in response to pharmaceutical pollution in a wastewater-impacted stream (Corcoll et al., 2014). Further research to identify bacterial taxa that thrive in multi-pollutant conditions may help identify biofilm responses to the variety of contaminants associated with urbanization (Walsh et al., 2005).

Last, we were most surprised to find that in contrast to our hypothesis, prior exposure to nutrient and pharmaceutical pollution in urban streams did not dampen the effects of our contaminant treatments on biofilm bacteria. Instead, pharmaceutical additions elevated bacterial core richness by 37% at urban sites and only 6% at montane sites. Existing nutrient and pharmaceutical pollution may have acted as an environmental filter selecting for taxa ready to capitalize on caffeine and diphenhydramine. Similarly, experimental additions of the antibiotic ciprofloxacin had an immense effect on community composition of urban biofilms in streams along a suburban to urban gradient in Maryland, United States (Rosi et al., 2018), with ciprofloxacin increasing the abundance of *Pedobacter* spp., a genus known for its ability to degrade unusual compounds (Kämpfer, 2010), and an unclassified genus from the family Bradyrhizobiaceae, which included several strains that are able to metabolize halogenated aromatic compounds (Kamal and Wyndham, 1990; Van der Woude et al., 1994; Egland et al., 2001). Thus, urbanization may create a predictable occurrence of pharmaceuticals entering streams that allows contaminant-tolerant bacteria to colonize biofilms.

## Conclusion

Both nutrients and pharmaceuticals structured biofilm bacterial cores at montane and urban sites, albeit in distinctly different ways. While N, P, and Fe additions enhanced the dominance of already abundant, copiotrophic heterotrophs, such as Pseudomonadaceae and Comamonadaceae, caffeine and diphenhydramine additions supported rich bacterial cores containing unique taxa associated with contaminant-degradation. In addition, nutrient plus pharmaceutical bacterial cores contained unique taxa, many of which belonged to Oscillatoriales, suggesting that these taxa possess superior competitive abilities to scavenge nutrients and tolerate and/or capitalize on multiple contaminants at a single time. Surprisingly, exposure to ambient nutrient and pharmaceutical pollution failed to reduce the effects of these contaminants on bacterial cores at urban sites, highlighting the potential for pollution to act as an environmental filter selecting for taxa that may readily respond to urban water conditions.

## Data Availability Statement

The datasets generated for this study can be found in the datasets generated and analyzed for this study are publicly available online at the Hydroshare database: http://www.hydroshare.org/resource/1f9c2b8d4fbd4c849e8e492cb0fd9198.

## Author Contributions

EO, MB, ER, DL, and ZA contributed to the study design. EO and DL carried out the experiment and lab work. EO and TS carried out the analyses. EO, MB, and ZA wrote the manuscript. All authors substantially contributed to commenting and revising it.

## Conflict of Interest

The authors declare that the research was conducted in the absence of any commercial or financial relationships that could be construed as a potential conflict of interest.

## Footnotes

1 https://dnasc.byu.edu
