# Supplementary material for: Nutrients and Pharmaceuticals Structure Bacterial Core Communities in Urban and Montane Stream Biofilms
Source: Front Microbiol. 2020 Oct 15;11:526545. doi: 10.3389/fmicb.2020.526545 (PMC7593328; doi:10.3389/fmicb.2020.526545)
Supplement: Supplementary file 1 [file Data_Sheet_1.docx]

**SUPPLEMENTAL MATERIAL**


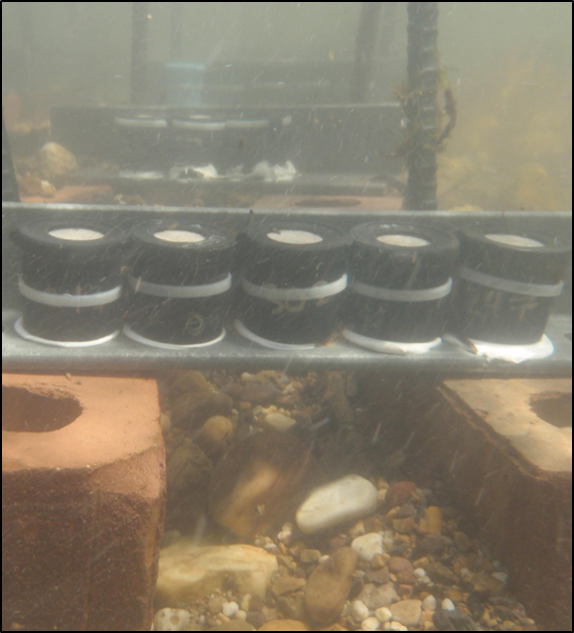


**Supplemental Figure 1.** Photo of contaminant exposure substrates (CES) deployed in a stream. Each CES consists of a small plastic cup (black cups in photo) filled with unamended or contaminant-amended agar. A porous substrate capping the agar (white glass discs in photo) serves as a platform for biofilm colonization. Biofilms growing on the porous substrates are exposed to contaminants which diffuse out of the agar. The front of the photo shows five CES fastened with zip-ties to a L-bar which is anchored to the stream bottom with bricks.

**Supplemental Table 1.** Number of OTUs in the total bacterial community and bacterial core communities for eight treatment by land-use combinations (i.e., control, nutrient, pharmaceutical, and nutrient plus pharmaceutical bacterial core communities in montane and urban streams). Core communities (cores) are defined as all OTUs present in at least 75% of samples in a given treatment by land-use combination. Treatment abbreviations are as follows: nutrient addition (Nu), pharmaceutical addition (Ph), and nutrient plus pharmaceutical addition (NuPh). Total number of OTUs is the number of unique OTUs in each community/core. Percent abundant and rare are the number of unique abundant (≥0.1% relative abundance) and rare (<0.1% relative abundance) OTUs in each community/core divided by the total number of unique OTUs in each community/core.

| **Community/Core** | **Total number of OTUs** | **% abundant OTUs** | **% rare OTUs** |
| --- | --- | --- | --- |
| Total community | 18984 | 0.81 | 99 |
|  |  |  |  |
| Core |  |  |  |
| Control montane | 435 | 29 | 71 |
| Nu montane | 298 | 42 | 58 |
| Ph montane | 455 | 28 | 72 |
| NuPh montane | 350 | 37 | 63 |
| *Mean of montane cores (SE)* | *385 (36.7)* | *34 (3.4)* | *66 (3.4)* |
|  |  |  |  |
| Control urban | 586 | 22 | 78 |
| Nu urban | 505 | 25 | 75 |
| Ph urban | 775 | 18 | 82 |
| NuPh urban | 439 | 29 | 71 |
| *Mean of urban cores (SE)* | *576 (72.8)* | *24 (2.4)* | *77 (2.4)* |
